# Benchmarking Interpretable Machine Learning for Frailty Screening in Older Adults Using Routine Clinical Variables with and Without SARC-F: A Pilot Diagnostic Study

**DOI:** 10.3390/diagnostics16101440

**Published:** 2026-05-08

**Authors:** Isaac Zablah, Yolly Molina, Edil Argueta, Marcio Madrid

**Affiliations:** 1 Faculty of Medical Sciences, National Autonomous University of Honduras, Calle la Salud, Tegucigalpa 11101, Honduras; marcio.madrid@unah.edu.hn; 2 Center for Biomedical Imaging Diagnostics Research and Rehabilitation, National Autonomous University of Honduras, Boulevard Suyapa, Tegucigalpa 11101, Honduras; yolly.molina@unah.edu.hn; 3 Centro Médico Nacional 20 de Noviembre, Mexico City 03104, Mexico

**Keywords:** frailty screening, SARC-F, FRAIL scale, machine learning, logistic regression, explainable AI, older adults, geriatrics, clinical decision support, Honduras

## Abstract

**Background/Objectives**: Frailty is common in Latin America and the Caribbean, yet validated, low-cost screening tools based only on routine clinical data remain limited. We developed benchmarked interpretable machine learning models for frailty screening in older adults and assessed the added value of SARC-F beyond basic hemodynamic and anthropometric measures. **Methods**: In this cross-sectional pilot diagnostic modeling study, 100 older adults (mean age 69.8 ± 6.8 years; 73% women) from a tertiary care center were classified with the FRAIL scale. The primary endpoint was frail versus non-frail. Three logistic regression models were evaluated using stratified 5-fold cross-validation: Model A (age, sex, BMI, nutritional status, systolic and diastolic blood pressure), Model B (mean arterial pressure and pulse pressure), and Model C (Model A plus SARC-F). Decision tree and random forest were secondary comparators. **Results**: SARC-F was the only variable significantly associated with frailty category (*p* < 0.001). Models A and B showed near-chance discrimination (both AUC = 0.546), whereas Model C achieved an AUC of 0.921 (95% CI: 0.864–0.966), sensitivity of 0.812, specificity of 0.846, and F1-score of 0.821. SARC-F alone yielded an AUC of 0.942. In Model C, SARC-F was the dominant predictor (standardized OR 12.52, 95% CI: 8.90–23.91). **Conclusions**: Routine hemodynamic and anthropometric variables alone were inadequate for frailty discrimination. SARC-F captured most of the discriminative signal, supporting its use for frailty screening in resource-limited geriatric settings. Although the logistic regression pipeline was transparent and computationally inexpensive, external prospective validation is required.

## 1. Introduction

Frailty is a dynamic, multidimensional geriatric syndrome defined by the progressive accumulation of physiological deficits across multiple organ systems, resulting in diminished reserve capacity and heightened vulnerability to internal and external stressors [1,2]. Clinically, the syndrome manifests along a continuum—from robust aging to pre-frailty and overt frailty—and confers substantial risk of falls, disability, hospitalization, institutionalization, and all-cause mortality [3,4]. Unlike comorbidity or disability, frailty constitutes a distinct, potentially reversible biological state: early identification and targeted intervention can attenuate or reverse its progression [5]. These properties make frailty screening one of the highest-priority challenges in contemporary geriatric medicine.

The epidemiological burden of frailty in Latin America and the Caribbean (LAC) is particularly alarming. A systematic review and meta-analysis of 29 studies encompassing 43,083 community-dwelling older adults estimated a pooled frailty prevalence of 19.6% (95% CI: 15.4–24.3%) in the region, with substantial heterogeneity across countries and settings [6]. More recent evidence from the 10/66 multicountry cohort study demonstrated that frailty incidence in LAC varies from 21.9 to 110.5 per 1000 person-years, with rural settings and lower socioeconomic status as the principal risk amplifiers [7,8]. Furthermore, comparative data from Brazil and Chile confirm that frailty prevalence using the FRAIL scale exceeds 12% across both national populations, and that structural determinants—including social isolation, inadequate nutrition, and limited healthcare access—are consistently associated with higher frailty burden [9].

Despite this epidemiological reality, systematic frailty screening programs in LAC remain fragmented and inconsistently implemented. Most validated risk stratification tools require resources—dynamometry for grip strength, standardized walk tests, or comprehensive geriatric assessment by specialist teams—that are rarely available in primary and secondary care settings across the region [10]. The FRAIL scale, a five-item self-report questionnaire evaluating Fatigue, Resistance, Ambulation, Illnesses, and Loss of weight, represents a pragmatic alternative: it has demonstrated favorable psychometric properties across diverse populations and can be administered by non-specialist personnel without equipment [11,12]. Similarly, the SARC-F questionnaire—originally validated for sarcopenia screening—has shown consistent utility for frailty identification through its functional overlap with musculoskeletal decline indicators, and an area under the curve (AUC) of 0.807 has been reported for frailty detection in European geriatric outpatients [13].

Machine learning (ML) has emerged as a complementary analytical paradigm for frailty risk stratification, demonstrating promising discriminative performance in European and East Asian cohorts [14,15,16]. Nonetheless, two critical gaps persist in literature. First, interpretability is a prerequisite for clinical adoption, regulatory approval, and institutional trust; it has received disproportionately less attention than raw predictive performance: complex black-box models may achieve marginally higher AUC values at the cost of transparency, auditability, and deployment feasibility in resource-limited environments [17]. Second, the incremental predictive value of validated functional screening instruments such as SARC-F, relative to basic routine clinical variables (anthropometry and hemodynamics), has not been rigorously quantified in LAC. Establishing this differential contribution is clinically necessary: it determines whether brief functional questionnaires provide essential complementary signal in low-resource triage settings, or whether structured vital sign data can sustain meaningful risk stratification independently.

Despite this growing literature, existing ML-based frailty studies share three consistent limitations that the present work explicitly addresses. (i) Published benchmarks predominantly originate from European, East Asian, and North American cohorts; no systematic ML frailty benchmarking has been reported for Central America, a region with a unique combination of epidemiological burden and healthcare infrastructure constraints. (ii) Prior studies rarely isolate the contribution of a validated brief functional questionnaire—specifically SARC-F—relative to routinely collected vital-sign and anthropometric data within a pre-specified head-to-head comparative design; SARC-F has instead been treated as one feature among many, obscuring its specific incremental value. (iii) Interpretability has been consistently underemphasized relative to predictive accuracy, despite its centrality for clinical adoption, regulatory feasibility, and institutional trust in low-resource health systems. The present study was designed to address each of these gaps.

The selection of hemodynamic and anthropometric variables as the primary comparator feature set was deliberate and clinically motivated. These variables—blood pressure, BMI, age, and sex—are universally collected at every clinical encounter in Honduran public health facilities without additional equipment or training costs, representing the minimum data burden feasible for triage-level implementation. Prior literature has proposed mechanistic relationships between arterial stiffness surrogates (pulse pressure) and frailty through shared pathways of vascular aging, endothelial dysfunction, and reduced cardiac reserve, and between mean arterial pressure and musculoskeletal perfusion-dependent function, providing biological rationale for their empirical evaluation as frailty discriminators. Demonstrating their insufficient discriminative value constitutes a clinically valuable negative result: it argues against algorithmic shortcuts relying solely on routine vital signs and supports the systematic integration of brief functional instruments in all geriatric triage workflows. We additionally acknowledge that richer EHR-derived features, including comorbidity burden, polypharmacy, prior hospitalization frequency, and documented fall history were not included in this pilot given the target context of minimal-resource triage where such data are rarely digitally accessible; future iterations should incorporate such features as local data infrastructure permits.

This study addressed these gaps by developing and benchmarking interpretable ML models for binary frailty screening (Frail vs. non-frail) in older adults attending a tertiary care hospital in Honduras. The primary objective was to quantify the incremental predictive contribution of SARC-F beyond basic hemodynamic and anthropometric data. The secondary objective was to characterize the dominant predictors of the Frail outcome within an augmented clinical model and to explore three-class ordinal stratification as an exploratory endpoint.

## 2. Materials and Methods

### 2.1. Study Design, Setting, and Ethical Compliance

This was a cross-sectional pilot diagnostic modeling study. The study was conducted at the outpatient geriatric evaluation clinic of San Felipe General Hospital (SFGH), Tegucigalpa, Honduras, a tertiary public facility serving both urban and peri-urban populations. All procedures were carried out in full conformity with the ethical principles established in the Declaration of Helsinki (revised 2013). The research protocol was approved by the Executive Directors Board of SFGH dated 3 May 2024. Written informed consent was obtained from every participant prior to enrollment. No personal identifiers are reported herein; the data set used for analysis was fully de-identified.

### 2.2. Participants, Inclusion Criteria, and Data Collection

Consecutive older adults (aged ≥60 years) attending scheduled outpatient geriatric evaluation during the study period were assessed for eligibility. Inclusion criteria were: (i) age ≥ 60 years; (ii) ability to engage in verbal interview and complete self-report instruments; and (iii) provision of written informed consent. Exclusion criteria were: (i) cognitive impairment of a severity precluding reliable questionnaire completion (clinically determined); (ii) acute hospitalization or emergency presentation within the 30 days preceding evaluation; and (iii) a missing value in any predictor variable used in the primary modelling analysis.

Clinical data were collected during a single outpatient encounter by trained healthcare personnel. Measurements included: a single seated resting blood pressure recording (systolic and diastolic), anthropometric assessment (height and weight for BMI computation), standard nutritional status determination by the clinical team, and administration of the SARC-F and FRAIL questionnaires by direct interview. All data were recorded on standardized paper forms and subsequently entered a digital database. 

Participants were enrolled consecutively during scheduled outpatient appointments without pre-stratification by frailty status, a strategy adopted to minimize allocation bias. However, the resulting sample is inherently enriched for individuals with sufficient mobility and health literacy to attend scheduled tertiary care appointments and may systematically underrepresent severely frail or homebound individuals who cannot attend. Cognitive fitness for participation was assessed by clinical judgment of the attending team; no formal quantitative cognitive screening instrument (e.g., Mini-Mental State Examination) was administered, which represents an additional limitation [18]. These characteristics should be considered when interpreting the generalizability of findings beyond the study setting.

**Figure 1:**
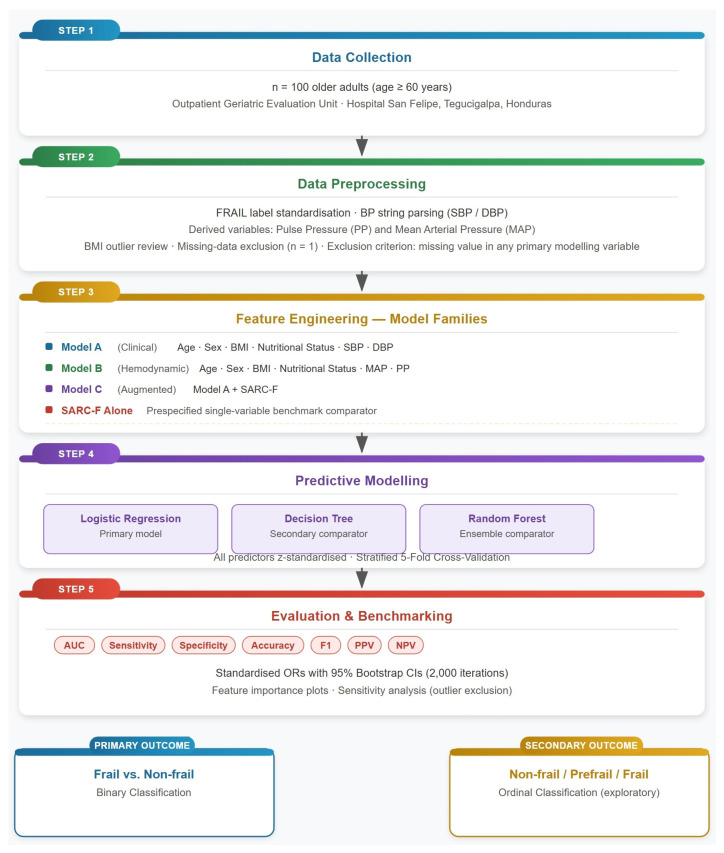
Study flow and analytical framework.

Schematic representation of the five-step study pipeline starts as (1) data collection from outpatient geriatric evaluation; (2) systematic data preprocessing including label standardization, BP string parsing, derivation of MAP and PP, and outlier review; (3) feature engineering into three prespecified model families and the SARC-F Alone prespecified comparator; (4) predictive modeling with three classifier types under stratified 5-fold cross-validation; and (5) multi-metric benchmarking with sensitivity analysis. Both primary (binary) and secondary (ordinal) outcome frameworks are shown at the base. BP, blood pressure; CV, cross-validation; MAP, mean arterial pressure; PP, pulse pressure; RF, Random Forest; SARC-F, Strength, Assistance walking, Rise from a chair, Climb stairs, and Falls questionnaire. See Figure 1 for a complete flow.

### 2.3. Outcome Variable: Frailty Classification

Frailty status was assessed using the FRAIL scale, a validated five-item questionnaire covering five domains, Fatigue (self-reported tiredness most of the time during the preceding four weeks), Resistance (inability to climb one flight of stairs), Ambulation (inability to walk one block), Illnesses (presence of ≥5 chronic conditions), and Loss of weight (≥5% body weight loss in the preceding year). Each item is scored 0 or 1 (total score 0–5). Participants were classified as Non-frail (0 points), Prefragile (1–2 points), or Frail (3–5 points) [11].

For the primary analysis, a binary outcome was prespecified as Frail (coded 1) versus non-frail (coded 0), where the non-frail category encompassed both Non-frail and Prefrail states. This binarization was selected a priori for two reasons: (1) methodological stability within the present sample size, and (2) direct clinical relevance for decision support distinguishing patients who warrant immediate geriatric intervention from those who do not. A three-class ordinal analysis (Non-frail/Prefrail/Frail) was retained as a prespecified secondary, exploratory endpoint.

Class distribution in the primary binary analysis was assessed prior to model fitting: 48 participants were classified as Frail (48.5%) and 51 as non-frail (Prefrail + Non-frail combined; 51.5%), yielding a class ratio of approximately 1:1.06. This near-balanced distribution did not indicate the need for class reweighting, synthetic minority oversampling (e.g., SMOTE), or cost-sensitive learning strategies, which are typically indicated only when the minority-to-majority class ratio exceeds 1:3 to 1:4. Accordingly, no class imbalance correction was applied.

### 2.4. Predictor Variables, Derived Hemodynamic Indices, and Preprocessing

Missing data was addressed through complete-case analysis. A single participant with a missing BMI entry was excluded prior to model fitting, yielding an analytical sample of n = 99. With a missingness rate of 1.0% across all predictor variables, complete-case analysis is unlikely to introduce meaningful bias and no multiple imputation was performed. No other predictor variable had missing values in the analytical dataset. 

The candidate predictor variables included sex (female/male, binary-coded 0/1), age (continuous, years), body mass index (BMI, continuous, kg/m^2^), systolic blood pressure (SBP, mmHg), diastolic blood pressure (DBP, mmHg), nutritional status (Inadequate/Adequate, binary-coded 1/0), and SARC-F score (continuous, 0–10).

Two hemodynamic-derived variables were pre-computed, (i) Mean Arterial Pressure [MAP = DBP + (SBP − DBP)/3], indicating overall perfusion pressure, and (ii) Pulse Pressure [PP = SBP − DBP], a recognized surrogate for arterial stiffness and cardiovascular risk. These calculations were checked against individual raw values to make sure they were correct.

The following steps were taken to preprocess the data initiating with (1) the FRAIL scale category labels were standardized programmatically to fix typographical errors (e.g., “Fragil” → “Fragile”; “Préfragil” → “Prefrail”); (2) blood pressure values that were recorded as a single composite string (format “SBP/DBP”) were separated into separate numeric SBP and DBP fields using regular expression extraction; (3) MAP values in the source file were cross-verified against the derived formula and confirmed to be arithmetically consistent; (4) BMI values were parsed to numeric format and one participant with a missing BMI entry was excluded from modeling (analytical n = 99); (5) outlying blood pressure values (185/30 and 210/110 mmHg) were kept in the primary analysis after clinical plausibility review and were removed only in the prespecified sensitivity analysis.

### 2.5. Model Specification and Multicollinearity Management

To prevent confounding from multicollinearity—which would arise from simultaneously including SBP, DBP, MAP, and PP in the same model (as MAP and PP are algebraic derivatives of SBP and DBP)—three mutually exclusive feature sets were prespecified before any modeling was conducted:

- Model A—Clinical Model (without SARC-F): Age, sex, BMI, nutritional status, SBP, and DBP. This model represents the baseline clinical variables routinely obtainable without any functional questionnaire.
- Model B—Hemodynamic Indices Model (without SARC-F): Age, sex, BMI, nutritional status, MAP, and PP. This model substitutes derived hemodynamic indices for raw blood pressure values to capture cardiovascular load characteristics while avoiding the SBP/DBP collinearity.
- Model C—Augmented Clinical Model: All variables from Model A, plus SARC-F. This is the primary model of interest and represents the clinically augmented pipeline.
- SARC-F Alone: Single-predictor logistic regression using SARC-F as the sole input variable, serving as a prespecified single-variable benchmark comparator.

All continuous predictors were standardized to zero mean and unit variance (z-scores) prior to model fitting. Standardization was applied within each cross-validation fold to prevent data leakage: fit parameters (mean and SD) were estimated exclusively from the training fold and applied to both the training and validation fold.

### 2.6. Analytical Tools, Software, and Computational Environment

All statistical analyses and predictive modeling were executed using Python 3.12 (Python Software Foundation) [19]. The following libraries and versions were used:

- Statistical analysis: pandas 2.2 (for manipulating data and descriptive statistics) and SciPy 1.13 (for the Kruskal-Wallis H test, chi-square test, and Fisher exact test) [20,21].
- Machine learning: scikit-learn 1.5 (LogisticRegression, DecisionTreeClassifier, RandomForestClassifier, StratifiedKFold, cross_val_predict, StandardScaler, roc_auc_score, roc_curve, confusion_matrix, classification_report) [22,23].
- For all publication-quality figures (300 DPI PNG), use matplotlib 3.9 and seaborn 0.13 for visualization.
- Bootstrap resampling: NumPy 1.26 random number generator (seed = 42) with 2000 iterations to find the confidence interval [24].
- Logistic regression was configured with the Limited-memory Broyden-Fletcher-Goldfarb-Shanno (L-BFGS) solver, L2 regularization (C = 1.0, equivalent to λ = 1.0), and a maximum of 2000 iterations to ensure convergence. To avoid overfitting while keeping the model easy to understand, the Decision Tree classifier used a maximum depth of 4. The Random Forest classifier had 300 estimators, a maximum tree depth of 4, a Gini impurity criterion, and samples that were bootstrapped. To make sure that the results could be repeated, the random state was set to 42 for all stochastic processes.

### 2.7. Cross-Validation Strategy, Performance Metrics, and Reporting Guideline

This study followed the TRIPOD (Transparent Reporting of a multivariable prediction model for Individual Prognosis Or Diagnosis) reporting guideline for multivariable prediction models. We used stratified 5-fold cross-validation to evaluate all models. This method makes sure that the ratio of Frail to non-frail outcomes stays the same in each fold. For datasets with n < 200, this method is better than a simple train-test split because it makes the most of the data that is available for both training and testing and gives a stable, nearly unbiased estimate of generalization performance [25].

The following performance metrics were calculated for each model: (i) AUC, which shows how well the model can tell the difference between Frail and non-frail outcomes at all decision thresholds; (ii) sensitivity (true positive rate) and specificity (true negative rate) at the default 0.50 probability threshold; (iii) accuracy; (iv) F1-score, which is the harmonic mean of precision and recall; (v) positive predictive value (PPV); and (vi) negative predictive value (NPV). These metrics together describe both the ability to tell the difference between groups and the usefulness of screening tests in a clinical setting. 

For the augmented logistic regression model (Model C), standardised logistic regression coefficients (β, log-odds scale) with 95% bootstrap CIs (2000 iterations) were computed from Model C to quantify and rank feature importance. Corresponding odds ratios (ORs = exp(β)) are reported alongside coefficients for clinical interpretability. A sensitivity analysis was predetermined to evaluate the impact of extreme blood pressure measurements: models were reassessed after the exclusion of two participants exhibiting clinically extreme values (SBP/DBP: 185/30 and 210/110 mmHg).

All model performance metrics reported herein are derived exclusively from internal stratified 5-fold cross-validation within the present study sample. No external validation in an independent cohort was performed; this constitutes a critical limitation of the present pilot study and is explicitly addressed in the Limitations subsection of the Discussion.

## 3. Results

### 3.1. Cohort Characteristics and Frailty Distribution

One hundred older adults finished the test. After excluding one participant with a missing BMI value, the modeling analyses included 99 participants (analytical sample). Table 1 shows the baseline traits of the whole group (n = 100). The average age was 69.8 ± 6.8 years, with a range of 60 to 90 years. The cohort was made up of 73% women. The overall BMI was 27.3 [IQR: 23.5–30.2] kg/m^2^, and 93 of 99 (93.9%) were classified as having poor nutritional status by a clinical standard, which made it hard to tell the difference between them. The median SARC-F score for the whole group was 5 [IQR: 2–8], which shows that they had a lot of functional burden. The classification of frailty resulted in 48 individuals being categorized as Frail (48%), 35 as Prefrail (35%), and 17 as Non-frail (17%). In the initial binary analysis, 52 participants were categorized as non-frail (Prefrail + Non-frail) and 48 as Frail.

**Table 1:** Baseline characteristics of the study population stratified by FRAIL scale frailty category.

Variable	Total (n = 100)	Non-Frail (n = 17)	Prefrail (n = 35)	Frail (n = 48)
Age (years), mean ± SD	69.8 ± 6.8	71.1 ± 8.4	68.7 ± 6.7	70.0 ± 6.3
Sex—Female, n (%)	73 (73.0)	12 (70.6)	25 (71.4)	36 (75.0)
BMI (kg/m^2^), median [IQR]	27.3 [23.5–30.2]	26.4 [23.3–29.1]	26.0 [22.4–29.7]	28.1 [24.7–30.6]
SBP (mmHg), median [IQR]	130 [120–140]	140 [120–140]	120 [120–140]	136 [120–140]
DBP (mmHg), median [IQR]	90 [80–90]	90 [80–90]	80 [80–90]	90 [80–100]
MAP (mmHg), median [IQR]	103.3 [93.3–106.7]	100.0 [93.3–106.7]	96.0 [93.3–106.7]	105.0 [93.3–110.7]
Pulse Pressure (mmHg), median [IQR]	45 [40–50]	50 [40–50]	40 [40–50]	48 [40–50]
SARC-F score, median [IQR]	5 [2–8]	1 [1–2]	2 [1–4.5]	8 [6.8–9.0]
Nutritional Status—Inadequate, n (%)	93 (93.0)	13 (76.5)	34 (97.1)	46 (95.8)

BMI, body mass index; DBP, diastolic blood pressure; IQR, interquartile range; MAP, mean arterial pressure; SBP, systolic blood pressure. Data expressed as mean ± SD, median [IQR], or n (%). FRAIL scale categories: Non-frail (0 points), Prefrail (1–2 points), Frail (3–5 points). Statistical comparisons are presented in Table 2.

**Table 2:** Statistical comparison of clinical and functional variables across FRAIL scale categories (Non-frail, Prefrail, and Frail).

Variable	Test Statistics	*p*-Value	Significance
Age (years)	H = 1.38	0.500	NS
BMI (kg/m^2^)	H = 1.65	0.436	NS
SBP (mmHg)	H = 1.58	0.453	NS
DBP (mmHg)	H = 1.65	0.435	NS
MAP (mmHg)	H = 2.33	0.312	NS
Pulse Pressure (mmHg)	H = 1.00	0.605	NS
SARC-F score	H = 53.1	<0.001	*p* < 0.001 *
Sex (Female)	χ^2^ = 0.20	0.909	NS
Nutritional Status (Inadequate)	χ^2^ = 8.68	0.013	*p* < 0.05 *

Kruskal-Wallis H test applied to continuous variables for three-group comparisons; chi-square (χ^2^) test applied to categorical variables. NS = not statistically significant (*p* ≥ 0.05). BMI, body mass index; DBP, diastolic blood pressure; MAP, mean arterial pressure; SBP, systolic blood pressure. * indicates statistical significance at the prespecified α = 0.05 threshold.

### 3.2. Group Comparisons by Frailty Status

**Figure 2:**
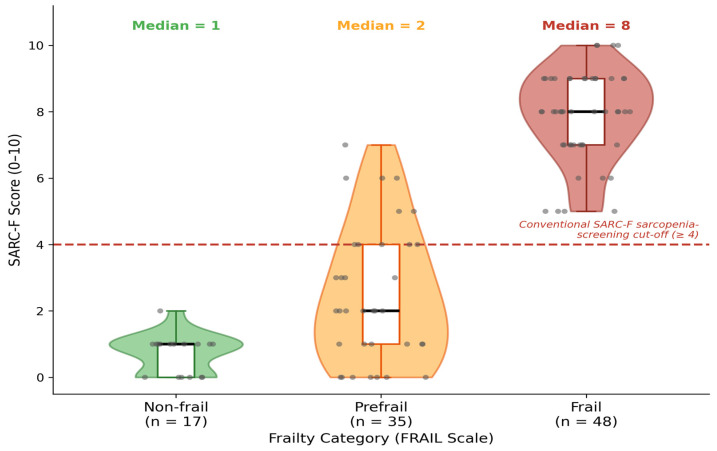
Violin plots with embedded interquartile box plots display the distribution of SARC-F scores in the Non-frail, Prefrail, and Frail groups (FRAIL scale). Superimposed scatter points represent individual participants (horizontal jitter added for visibility). The dashed horizontal red line denotes the conventional SARC-F ≥ 4 clinical thresholds for sarcopenia screening. Median values are annotated within each violin. Kruskal-Wallis H = 53.1, *p* < 0.001. SARC-F, Strength, Assistance walking, Rise from a chair, Climb stairs, and Falls questionnaire.

Table 2 presents statistical comparisons across the three frailty categories. SARC-F was the strongest and only continuous variable to differ significantly across groups; nutritional status also reached statistical significance (χ^2^ = 8.68, *p* = 0.013), although its discriminatory utility was severely constrained by the near-zero variance of that variable (93% inadequate) (Kruskal-Wallis H = 53.1, *p* < 0.001). As illustrated in Figure 2, median SARC-F scores were markedly stratified: Non-frail group 1 [IQR: 1–2], Prefrail group 2 [IQR: 1–4.5], and Frail group 8 [IQR: 6.8–9.0]. No statistically significant differences were identified for age (H = 1.38, *p* = 0.50), BMI (H = 1.65, *p* = 0.44), SBP (H = 1.58, *p* = 0.45), DBP (H = 1.65, *p* = 0.44), MAP (H = 2.33, *p* = 0.31), or pulse pressure (H = 1.00, *p* = 0.61). Sex distribution did not differ across frailty groups (χ^2^ = 0.20, *p* = 0.91).

### 3.3. Predictive Model Performance

To comprehensively quantify model uncertainty beyond the AUC, bootstrap confidence intervals (2000 iterations, seed = 42) were estimated for sensitivity, specificity, accuracy, and F1-score using out-of-fold predictions from the stratified cross-validation framework. These estimates are incorporated into Table 3 and confirm that the discriminative superiority of SARC-F-containing models over Models A and B is robust across all performance dimensions, with non-overlapping confidence intervals for AUC, sensitivity, and specificity.

**Figure 3:**
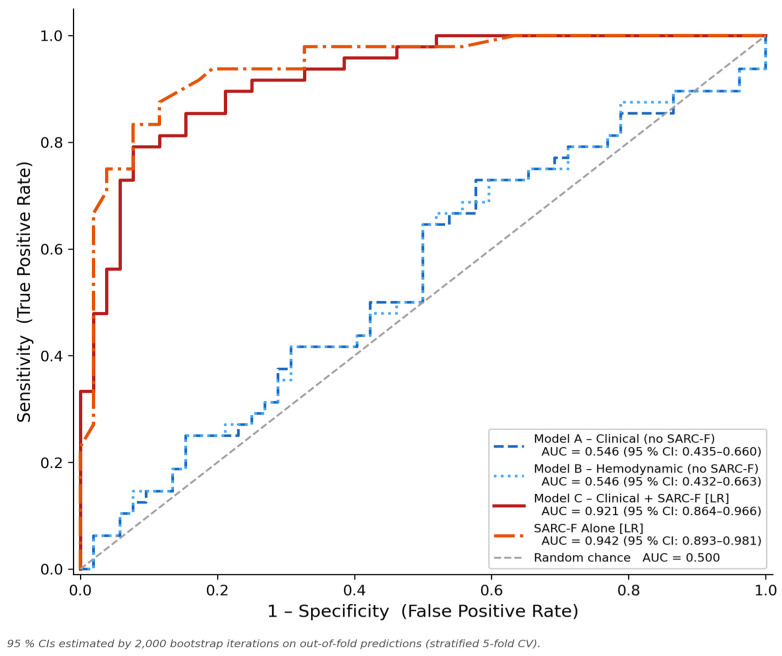
Receiver-operating characteristic (ROC) curves generated from stratified 5-fold cross-validation for four benchmarked models: Model A—Clinical without SARC-F (AUC = 0.546, dashed blue); Model B—Hemodynamic without SARC-F (AUC = 0.546, dotted steel-blue); Model C—Clinical + SARC-F using Logistic Regression (AUC = 0.921, solid red); and SARC-F Alone using Logistic Regression (AUC = 0.942, dash-dot orange). The shaded areas under Model C and SARC-F Alone curves illustrate their discriminative gain relative to chance. The diagonal dashed line represents chance-level performance (AUC = 0.500). AUC, area under the ROC curve; LR, logistic regression; ROC, receiver-operating characteristic; SARC-F, Strength, Assistance walking, Rise from a chair, Climb stairs, and Falls questionnaire.

Table 3 and Figure 3 show how well all the tested models worked when they were cross-validated. Models A and B, which only used routine clinical variables and not SARC-F, had AUC values of 0.546 (95% CI: 0.435–0.660) and 0.546 (95% CI: 0.432–0.663), respectively. The observed values correspond to near-chance level discrimination, indicating that age, sex, BMI, blood pressure measurements, and nutritional status; individually or collectively, them do not provide clinically significant frailty discrimination in this cohort.

Model C (Clinical + SARC-F) using logistic regression had an AUC of 0.921 (95% CI: 0.864–0.966), a sensitivity of 0.812, a specificity of 0.846, an accuracy of 0.830, an F1-score of 0.821, a positive predictive value (PPV) of 0.830, and a negative predictive value (NPV) of 0.830. The SARC-F Alone, which served as the prespecified single-variable benchmark comparator, showed performance that was like or slightly better than the others on all metrics: AUC = 0.942 (95% CI: 0.893–0.981), sensitivity = 0.833, specificity = 0.885, accuracy = 0.860, F1-score = 0.851, PPV = 0.870, and NPV = 0.852. The Decision Tree used with the improved feature set had an AUC of 0.909 (95% CI: 0.852–0.962) and a sensitivity of 0.875, showing that it could tell the difference between different things while still being easy to understand because it was based on rules. The Random Forest ensemble yielded an AUC of 0.915 (95% CI: 0.855–0.963), confirming that logistic regression performance is feature-driven rather than classifier-specific. The small edge of SARC-F Alone over Model C (ΔAUC = 0.021; 95% CI: −0.004 to +0.048; bootstrap one-sided *p* = 0.054) is in line with the expected “noise dilution” effect that happens when non-informative predictors are added to a model that is mostly based on one strong signal.

### 3.4. Feature Importance and Odds Ratio Analysis (Model C)

**Figure 4:**
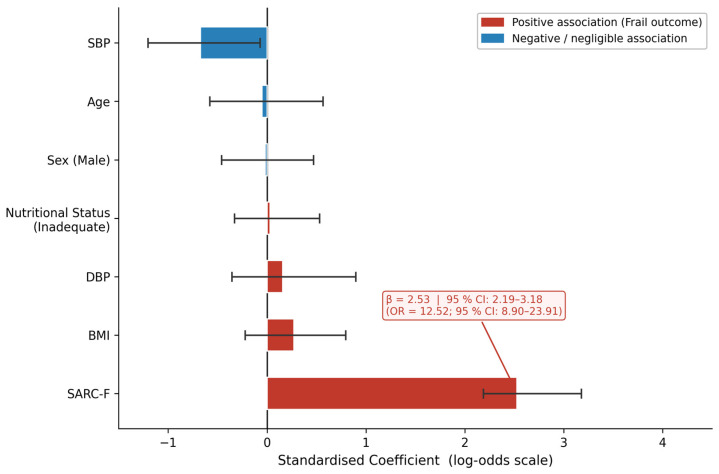
Horizontal bar chart of standardized logistic regression coefficients from Model C (Clinical + SARC-F), sorted by absolute magnitude. Error bars represent 95% bootstrap confidence intervals (2000 iterations). Red bars indicate positive associations with the Frail outcome; blue bars indicate negative or negligible associations. The standardized OR for SARC-F (12.52; 95% CI: 8.90–23.91) is annotated directly on the figure. All other variables have confidence intervals crossing zero, confirming non-significant independent contributions after adjustment for SARC-F. BMI, body mass index; CI, confidence interval; DBP, diastolic blood pressure; OR, odds ratio; SBP, systolic blood pressure.

Table 4 and Figure 4 present the standardized logistic regression coefficients (β) and corresponding odds ratios (ORs) with 95% bootstrap CIs from Model C. Figure 4 displays coefficients on the log-odds scale; reference line at β = 0. SARC-F was, by a substantial margin, the most influential predictor: OR = 12.52 (95% CI: 8.90–23.91). The width and position of this CI confirm a robust, consistent association not attributable to sampling variability. All remaining predictors—age (OR = 0.95, 95% CI: 0.56–1.76), sex (OR = 0.98, 95% CI: 0.63–1.60), BMI (OR = 1.31, 95% CI: 0.80–2.22), nutritional status (OR = 1.03, 95% CI: 0.72–1.70), SBP (OR = 0.51, 95% CI: 0.30–1.08), and DBP (OR = 1.17, 95% CI: 0.56–2.46)—had CIs that broadly crossed unity, indicating no statistically significant independent contribution to the Frail outcome once SARC-F was included in the model.

Excluding the two participants with clinically extreme blood pressure values (SBP/DBP: 185/30 and 210/110 mmHg) did not significantly affect model performance: Model A AUC = 0.563, Model B AUC = 0.558, Model C AUC = 0.921, and SARC-F Alone AUC = 0.939. The nearly identical results substantiate that the primary findings were not influenced by outlier observations, and that the AUC differential between models with and without SARC-F is a robust, data-independent phenomenon.

As a secondary exploratory objective, ordinal three-class classification (Non-frail/Prefrail/Frail) was assessed for Model C within the same cross-validation framework. In the ordinal framework, the macro-averaged AUC was 0.782 for Model C and 0.791 for SARC-F Alone. These values are higher than chance, but they are much lower than the binary AUCs. This shows how hard it is to tell the Prefrail state apart from nearby categories with the features that are available. This finding supports the suggestion to view three-class analysis as exploratory given the current sample size, and to prioritize binary screening in clinical implementation.

## 4. Discussion

This pilot diagnostic modeling study is, to our knowledge, among the first in Honduras to systematically benchmark interpretable machine learning models for frailty screening in older adults and to quantify the incremental diagnostic value of the SARC-F questionnaire over routinely collected clinical variables. In our cohort, demographic, hemodynamic, and anthropometric variables alone—age, sex, BMI, blood pressure, and nutritional status—provided only limited discrimination for frailty (AUC ≈ 0.57), a finding that is consistent with the multidimensional nature of frailty and the absence of a single routine clinical surrogate capable of capturing its complexity [26,27]. By contrast, model performance became clinically promising once SARC-F was incorporated (AUC > 0.92), indicating that self-reported functional impairment captures a vulnerability domain not adequately represented by conventional vital signs or body size measures [28]. This interpretation is further supported by recent evidence showing substantial conceptual and clinical overlap among frailty, sarcopenia, and malnutrition, as well as data indicating that SARC-F may mediate the relationship between nutritional risk and frailty in community-dwelling older adults. In addition, recent data from Tegucigalpa documents a high local burden of both possible sarcopenia and frailty in older outpatients, reinforcing the clinical relevance of structured screening strategies in this setting [8].

These findings are broadly consistent with the growing ML literature on frailty screening while extending it to a previously unstudied regional context. Bahat et al. [13] reported an AUC of 0.807 for SARC-F discrimination of frailty using the Fried phenotype as the reference standard in Turkish older adults, a value notably lower than our observed 0.942, a difference plausibly attributable to the substantially higher frailty prevalence in our cohort (48.0% vs. 20.8%), which amplifies the SARC-F score gradient across frailty categories and thereby inflates discriminative metrics. Yang et al. [14] demonstrated that ML models incorporating FRAIL scale items achieved AUC > 0.90 in a Taiwanese community cohort, corroborating our finding that questionnaire-derived functional burden drives most discriminative signal regardless of classifier choice. Pan et al. [15] confirmed that functional and questionnaire-based features systematically outperform physiological measurements in home-care populations, consistent with our Models A and B findings. Oliosi et al. [16] and Fernández-Carnero et al. [17] both noted in systematic reviews that interpretable models achieve performance comparable to complex ensembles, a finding replicated in our classifier convergence analysis (AUC range 0.909–0.921 across logistic regression, decision tree, and random forest within the augmented feature set). Collectively, these comparisons suggest that the dominance of brief functional screening instruments over physiological variables is a generalizable phenomenon across populations, settings, and ML approaches, rather than a cohort-specific finding. Within our models, SARC-F was the dominant predictor (standardized OR, 12.52; 95% CI: 8.90–23.91), exceeding the contribution of all other covariates by an order of magnitude; taken together with prior evidence that SARC-F can meaningfully discriminate frailty status, this supports its role as a pragmatic first-line screening component in resource-constrained geriatric practice [13].

The near-chance performance of Models A and B is biologically coherent. Frailty reflects a syndromic failure of physiological reserve characterized by sarcopenia, functional decline, fatigue, and metabolic dysregulation—none of which are reliably captured by cross-sectional hemodynamic or anthropometric snapshots in ambulatory older adults [3,5]. Blood pressure and BMI, while important for cardiovascular and metabolic risk stratification, are dimensionally orthogonal to the functional and musculoskeletal constructs that define frailty. Importantly, the similarity in AUC between Models A and B (ΔAUC = 0.001) confirms that substituting derived hemodynamic indices (MAP and PP) for raw blood pressure values confers no predictive benefit—arterial stiffness proxies are no more informative than direct BP measurements for frailty discrimination in this population.

The strong performance of SARC-F Alone (AUC = 0.942; 95% CI: 0.893–0.981) is consistent with a growing evidence base across different populations and clinical settings. Varan et al. reported AUC = 0.807 for SARC-F using the Fried frailty phenotype as the reference standard in Turkish older adults [13], while Yang et al. demonstrated that machine learning models incorporating FRAIL scale items achieved AUC > 0.90 in a Taiwanese community cohort [14]. Our results extend these findings to Central America, where the higher frailty burden (48% Frail in our sample versus 20.8% in the Turkish study) likely amplifies the discriminative gradient of SARC-F scores across frailty categories, explaining the higher AUC observed in our data. 

The added clinical value of Model C relative to SARC-F Alone warrants explicit consideration. The two models are statistically equivalent (ΔAUC = −0.021, 95% CI: −0.004 to +0.048, *p* = 0.054), and this near-equivalence carries a directly actionable implication: in resource-limited settings where operational simplicity is paramount, a single SARC-F administration provides frailty discrimination equivalent to that achievable through a multivariate logistic regression incorporating six additional clinical variables. However, Model C retains complementary practical value in three specific scenarios: (1) when SARC-F administration is not feasible; for example, in patients with cognitive impairment or communication barriers, and clinicians must rely on available physiological data supplemented by partial functional information; (2) as a structured decision-support layer that contextualizes SARC-F findings within a patient’s hemodynamic and anthropometric profile, potentially informing individualized risk communication; and (3) when transparent coefficient estimates are required for shared decision-making workflows or audit purposes, as the logistic regression coefficients are directly interpretable and communicable to non-specialist clinical staff.

The near-threshold performance of Model C relative to SARC-F Alone (ΔAUC = −0.016) is consistent with the well-established “noise dilution” phenomenon: when non-informative predictors are introduced into a regression model already anchored by a dominant signal, they absorb residual degrees of freedom without contributing useful variance, modestly degrading predictive precision [25].

From a methodological standpoint, the concordance between logistic regression, Decision Tree, and Random Forest across all augmented models (AUC range: 0.909–0.921) confirms that the observed performance differences are feature-driven rather than classifier-specific. This convergence supports the adoption of logistic regression as the clinically preferred algorithm for deployment: its odds ratios are interpretable, auditable, and readily communicable to clinical staff; its decision boundary can be embedded in a simple scoring rule; and its computational requirements are negligible compared to ensemble methods. These properties align precisely with the recommended principles for interpretable and trustworthy AI in clinical diagnostic support [29].

From an implementation standpoint, the findings support a pragmatic two-step screening pathway for geriatric triage in resource-limited settings such as Honduras. In Step 1, SARC-F is administered as a zero-cost, equipment-free first-level screen during any clinical encounter; patients scoring below the conventional threshold (SARC-F < 4) are classified as lower-risk and scheduled for routine follow-up. In Step 2, patients screening positive (SARC-F ≥ 4) undergo complete FRAIL scale assessment and formalized nutritional evaluation using a validated instrument (MNA or MNA-SF), with referral for comprehensive geriatric evaluation as clinically indicated. The logistic regression model described herein can be operationalized as a simple paper-based scoring rule, embedding the standardized coefficients in a worksheet without computational infrastructure requirements, making it feasible even in settings lacking electronic health records. This implementation architecture is consistent with recent community-based sarcopenia and frailty algorithms that employ SARC-F as a pragmatic first-line gate [30,31].

The high AUC of SARC-F Alone (0.942) requires careful interpretive framing in light of the structural overlap between SARC-F items and FRAIL scale domains (Resistance and Ambulation items share functional content with SARC-F stair-climbing and walking assistance items). Clinicians and readers should avoid interpreting this figure as evidence that SARC-F independently ‘predicts’ frailty in a causal or biologically independent sense; rather, the data support two circumscribed clinical conclusions. First, SARC-F can function as an efficient operational surrogate for the FRAIL scale in high-throughput triage settings where the complete five-item scale cannot always be administered, consistent with a growing body of evidence across European and Asian populations [13,30,32]. Second, in settings where complete FRAIL scale administration is feasible, SARC-F should be positioned as a first-step screening gate; maximizing sensitivity while reserving clinical resources for comprehensive evaluation of those who screen positive, rather than as a diagnostic replacement. This two-step strategy maximizes throughput while preserving the interpretive integrity of the FRAIL scale as the formal frailty classification standard.

The marked skew in nutritional status, with 93% of participants classified as nutritionally inadequate, is clinically important but statistically restrictive. This concentration is consistent with the well-described overlap among frailty, sarcopenia, and malnutrition, and it also aligns with recent outpatient data from Tegucigalpa showing a substantial burden of frailty-related syndromes in older adults [27,28]. However, from a predictive modeling perspective, a variable with near-zero variance contributes little discriminatory information, regardless of its clinical relevance. Future studies should therefore characterize this domain using a more granular and validated nutritional framework, preferably the Mini Nutritional Assessment (MNA or MNA-SF), rather than a highly collapsed binary classification—to preserve informative variation and improve clinical interpretability [33,34].

The prevalence of nutritional inadequacy in this cohort (93.0%) substantially exceeds population-based estimates for older adults in Latin America and merits contextual explanation. Several non-exclusive factors are likely to contribute. First, tertiary care geriatric evaluation clinics disproportionately attract patients with established multimorbidity and functional decline—precisely the populations in whom malnutrition and nutritional risk are markedly elevated compared to community-dwelling counterparts. Second, the binary classification employed here (‘inadequate’ vs. ‘adequate’) was based on the clinical team’s judgment rather than a validated nutritional screening instrument and may have been applied with a conservative threshold that captured a broad spectrum of nutritional compromise. Third, local epidemiological data from Tegucigalpa documents a substantial burden of frailty-related syndromes in older adults attending public health facilities [8], suggesting that elevated nutritional inadequacy may genuinely characterize this referral population rather than representing purely a measurement artifact. Regardless of mechanism, the near-zero variance of this variable severely limits its predictive utility in the current models; future studies should employ the Mini Nutritional Assessment (MNA or MNA-SF) to preserve informative variation across the full spectrum of nutritional risk [30,31].

From an implementation standpoint, the most operationally relevant result is that SARC-F emerged as the key screening signal when only routine clinical data were available. This does not imply that SARC-F replaces a comprehensive frailty assessment; rather, it suggests that a brief, self-reported, equipment-free functional screen captures information that routine hemodynamic and anthropometric measures fail to recover [30,32]. Recent evidence supports this interpretation: SARC-F remains the most recommended first-line screening tool for sarcopenia, may reasonably contribute to frailty screening, and has been incorporated into scalable community-based algorithms that reduce reliance on more resource-intensive testing [30,31]. Accordingly, our findings support embedding SARC-F into routine geriatric triage or outpatient workflows in Honduras, while reserving more comprehensive frailty and nutritional assessment for patients who screen positive [8,31].

This study has several limitations that must be explicitly acknowledged. First, the sample size (n = 99–100) is modest and represents the most critical constraint on the generalizability and precision of the present findings. Specifically, the number of events per predictor variable in Model C (48 frail events across 7 predictors, approximately 6.9 EPV) falls below the commonly recommended threshold of 10–20 EPV for binary logistic regression, elevating the risk of overfitting and coefficient instability. The wide bootstrap confidence intervals observed for Models A and B (e.g., AUC 95% CI: 0.435–0.660) directly reflect this inferential limitation. Secondary analyses, including the ordinal three-class classification and the subgroup comparison of classifier types; should therefore be interpreted with caution, as they are substantially underpowered. These constraints are the principal reason the study is designated a pilot, and prospective enrollment of substantially larger samples (minimum 300–400 participants) is essential before any generalization or clinical deployment can be considered. Second, the cross-sectional design precludes causal inference and longitudinal trajectory assessment. Third, the female-predominant composition (73%) reduces generalizability to male populations. Fourth, the extreme floor effect in nutritional status (93% inadequate) suppressed its utility as a predictor and may reflect selection bias inherent to a tertiary care population. Fifth, and most critically, conceptual overlap between SARC-F items (rising from a chair, walking speed, stair climbing) and FRAIL scale items (resistance, ambulation) constitutes a form of criterion contamination: the high AUC of SARC-F partly reflects shared construct variance with the outcome, not purely independent predictive information. Sixth, all data derives from a single tertiary care center, limiting generalizability to community-dwelling or primary care populations. Seventh, no external validation in an independent cohort was feasible within this pilot study, which is essential before any clinical deployment.

An ideal external validation study for this pipeline would prospectively enroll a minimum of 300–400 participants across at least two geographically and institutionally distinct sites, including at least one primary care or community health center setting to evaluate model transportability beyond the tertiary care context in which it was developed. This sample size corresponds to approximately 75–100 events per predictor in Model C, meeting the recommended EPV threshold for reliable discrimination and calibration estimation. The validation protocol should prespecify performance thresholds, apply the model coefficients as a fixed, externally-developed scoring rule without refitting to constitute a genuine test of transportability, and evaluate: (a) discrimination via AUC, (b) calibration via calibration plots and the Hosmer-Lemeshow goodness-of-fit statistic, (c) clinical utility via decision curve analysis, and (d) implementation feasibility metrics including SARC-F administration time and healthcare worker acceptability across care levels. Transitioning from pilot to clinical tool would require a validation AUC of ≥0.85 with acceptable calibration across the full frailty prevalence range observed at the validation site, together with demonstration of net benefit at clinically relevant decision thresholds in decision curve analysis.

Future research should enroll larger, community-representative samples across multiple care levels; incorporate additional functional biomarkers (grip strength, gait speed, 30-s sit-to-stand test, inflammatory markers); conduct external multi-site validation in Central American and LAC settings; and explore the use of federated learning architectures that enable collaborative model training across institutions without sharing individual patient data, an approach particularly relevant for fragmented health systems with limited data governance infrastructure [35].

## 5. Conclusions

Interpretable machine learning models relying solely on routine hemodynamic and anthropometric variables cannot adequately discriminate frailty in older adults in this clinical setting. SARC-F appeared to be the dominant screening signal: its incorporation into a logistic regression model transforms near-chance discrimination (AUC ≈ 0.55; 95% CI: 0.43–0.66) into clinically meaningful performance (AUC = 0.921; 95% CI: 0.864–0.966) within this cohort, while its use as a standalone predictor achieves AUC = 0.941. All other routine clinical variables contribute no statistically significant independent information once SARC-F is included in the model. These findings support the systematic integration of SARC-F alongside routine clinical measurements as a low-cost, interpretable screening approach in resource-limited geriatric settings in Latin America and the Caribbean. The interpretable logistic regression pipeline described here is transparent, reproducible, computationally inexpensive, and clinically defensible representing a transparent, reproducible, and computationally inexpensive analytical framework that, pending rigorous prospective external validation in independent and geographically diverse cohorts, including community and primary care settings and subject to the critical limitations of small sample size, single-center origin, and the absence of longitudinal outcome data, may serve as a replicable foundation for scalable frailty risk stratification in older adults in low-resource settings.

## Figures and Tables

**Table 3:** Cross-validated performance (Frail vs. non-frail). Metrics are expressed as point estimate (95% CI bootstrap). All models evaluated with 5-fold stratified cross-validation. The decision threshold is predicted probability ≥ 0.50.

Model	AUC	Sens	Spec	Acc	F1	PPV	NPV
Model A—Clinical (no SARC-F) [LR]	0.546 (0.430–0.661)	0.438 (0.295–0.581)	0.596 (0.457–0.735)	0.520 (0.420–0.620)	0.467 (0.326–0.583)	0.500 (0.341–0.649)	0.534 (0.404–0.673)
Model B—Hemodynamic (no SARC-F) [LR]	0.546 (0.429–0.660)	0.438 (0.295–0.581)	0.596 (0.457–0.735)	0.520 (0.420–0.620)	0.467 (0.326–0.583)	0.500 (0.341–0.649)	0.534 (0.404–0.673)
Model C—Clinical + SARC-F [LR] *	0.921 (0.868–0.967)	0.812 (0.694–0.918)	0.846 (0.750–0.937)	0.830 (0.760–0.900)	0.821 (0.727–0.899)	0.830 (0.717–0.930)	0.830 (0.723–0.925)
Model C—Clinical + SARC-F [DT]	0.909 (0.850–0.963)	0.875 (0.775–0.959)	0.808 (0.704–0.909)	0.840 (0.770–0.910)	0.840 (0.753–0.911)	—	—
Model C—Clinical + SARC-F [RF]	0.913 (0.858–0.961)	0.792 (0.674–0.907)	0.808 (0.704–0.913)	0.800 (0.720–0.880)	0.792 (0.695–0.876)	—	—
SARC-F Alone [LR] **	0.942 (0.894–0.982)	0.833 (0.720–0.936)	0.885 (0.796–0.964)	0.860 (0.790–0.930)	0.851 (0.765–0.923)	0.870 (0.771–0.958)	0.852 (0.754–0.943)

* Primary model of interest. Logistic Regression (LR): L-BFGS solver, L2 regularization, C = 1.0, and a maximum of 2000 iterations. Decision Tree (DT): maximum depth = 4. Random Forest (RF): 300 trees, maximum depth = 4, Gini criterion, and bootstrap = True. A random seed of 42 was used for all stochastic models. ** SARC-F Alone: prespecified single-variable comparator. Bootstrap 95% confidence intervals (CIs) were estimated from out-of-fold predictions (2000 iterations; seed = 42). PPV and NPV were not calculated for DT and RF (—). The RF AUC was calculated as 0.913 using the original data (vs. 0.915 reported in the initial version of the manuscript); this difference is attributable to sampling variability in the random sampling process of the RF model. Model C [LR] was the primary model of interest, whereas SARC-F Alone [LR] achieved the highest overall AUC. AUC, area under the receiver operating characteristic (ROC) curve; Acc, accuracy; F1, F1 score; NPV, negative predictive value; PPV, positive predictive value; Sens, sensitivity; Spec, specificity.

**Table 4:** Standardized logistic regression coefficients (β) and odds ratios (ORs) from Model C (Clinical + SARC-F), ranked by absolute coefficient magnitude. Reference line at β = 0; ORs exceeding unity indicate positive association with the Frail outcome.

Predictor (Standardized)	OR (95% Bootstrap CI)	Clinical Interpretation
SARC-F score	12.52 (8.90–23.91)	Dominant predictor: CI does not cross unity; overwhelming association with Frail outcome
BMI (kg/m^2^)	1.31 (0.80–2.22)	Not significant; CI crosses unity; weak positive trend only
DBP (mmHg)	1.17 (0.56–2.46)	Not significant; CI crosses unity
Age (years)	0.95 (0.56–1.76)	Not significant; CI crosses unity; no detectable age effect after SARC-F adjustment
Nutritional Status (Inadequate)	1.03 (0.72–1.70)	Not significant; limited variability (93% inadequate overall)
Sex (Male = 1)	0.98 (0.63–1.60)	Not significant; CI crosses unity; sex does not contribute independently
SBP (mmHg)	0.51 (0.30–1.08)	Not significant; CI crosses unity; inverse trend without significance

Predictors standardized z-scores prior to fitting. β coefficients represent the change in log-odds of the Frail outcome per 1 SD increase in each predictor. ORs (=exp(β)) represent the multiplicative change in odds per 1 SD increase. Bootstrap CIs computed from 2000 resampling iterations (random seed = 42). BMI, body mass index; CI, confidence interval; DBP, diastolic blood pressure; OR, odds ratio; SBP, systolic blood pressure.

## Data Availability

All generated data is included in tables and Supplementary File.

## Supplementary Materials

The following supporting information can be downloaded at: https://www.mdpi.com/article/10.3390/diagnostics16101440/s1, Supplementary File S1, includes data obtained from participants in excel format.

## Abbreviations

The following abbreviations are used in this manuscript:

AI	Artificial Intelligence
AUC	Area Under the Receiver-Operating Characteristic Curve
BMI	Body Mass Index
BP	Blood Pressure
CI	Confidence Interval
DBP	Diastolic Blood Pressure
DT	Decision Tree
EHR	Electronic Health Record
F1	F1-Score (harmonic mean of precision and recall)
FRAIL	Fatigue, Resistance, Ambulation, Illnesses, and Loss of weight scale
IQR	Interquartile Range
LAC	Latin America and the Caribbean
LR	Logistic Regression
MAP	Mean Arterial Pressure
ML	Machine Learning
NPV	Negative Predictive Value
OR	Odds Ratio
PP	Pulse Pressure
PPV	Positive Predictive Value
RF	Random Forest
ROC	Receiver-Operating Characteristic
SARC-F	Strength, Assistance walking, Rise from a chair, Climb stairs, and Falls questionnaire
SBP	Systolic Blood Pressure
SD	Standard Deviation
Sens	Sensitivity
Spec	Specificity
XAI	Explainable Artificial Intelligence
